# P-1838. The human Herpesvirus dUTPases of EBV and HSV-1 may contribute to neurological disorders by altering the integrity of the blood brain barrier and promoting a pro-inflammatory microenvironment

**DOI:** 10.1093/ofid/ofae631.2001

**Published:** 2025-01-29

**Authors:** Irene Mena-Palomo, Victor Martinez, Irene Munk Perdersen, Maria Ariza

**Affiliations:** The Ohio State University Wexner Medical Center, Columbus, Ohio; The Scintillon Research Institute, San Diego, California; The Scintillon Research Institute, San Diego, California; The Ohio State University Wexner Medical Center, Columbus, Ohio

## Abstract

**Background:**

The global prevalence of Herpes Simplex Virus 1 (HSV-1) and Epstein-Barr Virus (EBV) ranges from 60% to >90%. Some herpesviruses have been linked to neurological diseases including Alzheimer’s disease (AD) and one common feature to these pathologies is disruption of the blood-brain barrier (BBB). The BBB is a key structure responsible for protecting the brain from toxic substances due to its high impermeability and providing energy which is critical for proper brain function. While disruption of the BBB is a hallmark of numerous neurological disorders, the mechanisms underlying herpesvirus-induced BBB dysfunction remain elusive.

Herpesvirus dUTPases encoded by EBV and HSV-1 alter blood brain barrier integrity

**Figure:**
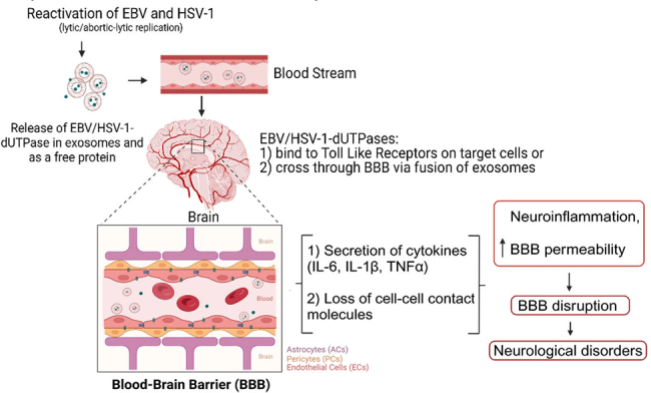

Stress, aging as well as other environmental and genetic factors can reactivate human herpesviruses to undergo lytic or abortive-lytic replication, which results in the release of viral dUTPases as a free protein or in exosomes. dUTPase-containing exosomes can travel to the brain via the bloodstream and target cells at the blood brain barrier, triggering the release of pro-inflammatory cytokines and disturbing the structural and functional properties of cell-cell contact molecules. Altogether, the herpesviruses dUTPases can contribute to neurological disorders by increasing blood brain barrier permeability and promoting neuroinflammation.

**Methods:**

In this study we investigated whether a viral protein of EBV and HSV-1 called dUTPase can alter BBB integrity using AD clinical samples, in vitro and 3D perfused hiPSC-derived BBB/NVU models, in combination with a multi-system analysis approach that included, trans-endothelial electrical resistance (TEER), confocal imaging, ELISA, proteome array and qRT-PCR/RNAseq.

**Results:**

Exposure of 3D BBB to HSV-1 or EBV dUTPases resulted in significant BBB permeability, reduced TEER, and loss of functional tight junction protein (TJP; ZO-1 and Claudin-5) structural and functional properties. Both dUTPases significantly increased the expression and/or secretion of IL-1β, IL-6 and TNF-α in immortalized human cerebral microvascular endothelial cells, astrocytes and pericytes. A modest but significant increase in IL-1β and IL-6 mRNA was also observed in dUTPase-treated astrocytes. These cytokines are elevated in patients with AD and are known to disrupt BBB integrity and induce neuroinflammation.

**Conclusion:**

In conclusion, this study provides compelling evidence that EBV/HSV-1 dUTPases have the potential to disrupt the BBB and foster a pro-inflammatory environment in the brain. Thus, it highlights a novel mechanism linking common herpesviruses to brain pathologies.

**Disclosures:**

**All Authors**: No reported disclosures

